# Sorafenib suppresses the epithelial-mesenchymal transition of hepatocellular carcinoma cells after insufficient radiofrequency ablation

**DOI:** 10.1186/s12885-015-1949-7

**Published:** 2015-11-30

**Authors:** Shuying Dong, Jian Kong, Fandong Kong, Jinge Kong, Jun Gao, Liang Ji, Bing Pan, Lian Chen, Lemin Zheng, Wenbing Sun

**Affiliations:** 1 Department of Hepatobiliary Surgery, Beijing Chao-yang Hospital, Capital Medical University, Beijing, 100043 China; 2 School of Basic Medical Sciences, and Key Laboratory of Molecular Cardiovascular Sciences of Ministry of Education, The Institute of Cardiovascular Sciences and Institute of Systems Biomedicine, Peking University Health Science Center, Beijing, 100191 China; 3 Department of Neurobiology, School of Basic Medical Sciences, The Neuroscience Research Institute, Peking University, Beijing, 100191 China; 4 Key Laboratory for Neuroscience, Ministry of Education/National Health and Family Planning Commission, Peking University, Beijing, 100191 China; 5 The 8th Department of Orthopaedics, Affiliated Mindong Hospital of Fujian Medical University, Fujian, 355000 China

**Keywords:** Hepatocellular carcinoma, Insufficient radiofrequency ablation, Epithelial-mesenchymal transition, Sorafenib

## Abstract

**Background:**

Epithelial-mesenchymal transition (EMT) played an important role in the progression of hepatocellular carcinoma (HCC) after insufficient radiofrequency ablation (RFA). However, whether sorafenib could be used to suppress the EMT of HCC after insufficient RFA and further prevent the progression of residual HCC remains poorly unknown.

**Methods:**

Insufficient RFA was simulated using a water bath (47 °C 5, 10, 15, 20 and 25 min gradually). MTT assay and transwell assay were used to evaluate the effects of sorafenib on viability, migration and invasion of HepG2 and SMMC7721 cells after insufficient RFA *in vitro*. After insufficient RFA, the molecular changes in HCC cells with the treatment of sorafeinb were evaluated using western blot and ELISAs. An ectopic nude mice model was used to evaluate the effect of sorafenib on the growth of HepG2 cells *in vivo* after insufficient RFA.

**Results:**

HepG2 and SMMC7721 cells after insufficient RFA (named as HepG2-H and SMMC7721-H) exhibited enhanced viability, migration and invasion *in vitro*. Sorafenib inhibited the enhanced viability, migration and invasion of HepG2 and SMMC7721 cells after insufficient RFA. Molecular changes of EMT were observed in HepG2-H and SMMC7721-H cells. Sorafenib inhibited the EMT of HepG2-H and SMMC7721-H cells. HepG2-H cells also exhibited larger tumor size *in vivo*. Higher expression of PCNA, Ki67, N-cadherin, MMP-2 and MMP-9, was also observed in HepG2-H tumors. Sorafenib blocked the enhanced growth of HepG2 cells *in vivo* after insufficient RFA.

**Conclusions:**

Sorafenib inhibited the EMT of HCC cells after insufficient RFA, and may be used to prevent the progression of HCC after RFA.

**Electronic supplementary material:**

The online version of this article (doi:10.1186/s12885-015-1949-7) contains supplementary material, which is available to authorized users.

## Background

Hepatocellular carcinoma (HCC) is the fifth most common tumor worldwide and is the third most common cause of cancer-related death [1]. Radiofrequency ablation (RFA) is emerging as an effective local treatment for curative intent in patients with cirrhosis and HCC smaller than 3 cm in diameter [2]. However, the major problem with RFA is its difficulty in achieving complete tumor destruction [3], and several cases of rapid and aggressive recurrence of HCC after RFA have been reported [4–6].

Several mechanisms have been proposed to explain the phenomenon of progression of HCC after insufficient RFA [7–12]. Epithelial-mesenchymal transition (EMT) is a complex process, involving dissolution of cell-cell junctions and loss of apical-basolateral polarity, resulting in transition of epithelial cells into migratory mesenchymal cells with invasive properties [13]. Yoshida et al. reported that sublethal heat treatment skews HCC cells toward EMT and transforms them to a progenitor-like, highly proliferative cellular phenotype *in vitro* and *in vivo*, which driven significantly by p46Shc-ERK1/2, and suboptimal RFA accelerates HCC growth and spread by transiently inducing an EMT-like, more aggressive cellular phenotype [12]. Our previous study established a model simulating insufficient RFA *in vitro* and showed that insufficient RFA could directly promote the invasiveness and metastasis of HCC cells and the EMT of HCC cells through Akt and ERK signaling pathways [11]. However, there are no researches about exploring effectual approaches to prevent the progression of HCC after insufficient RFA.

Sorafenib is the first and only molecular targeted therapy approved for use in HCC by the U.S. Food and Drug Administration in 2007. Currently, sorafenib is used as a standard treatment for patients with advanced HCC. Sorafenib inhibited the EMT induced by transforming growth factor β1 in mouse hepatocytes and hepatocyte growth factor in HCC [14, 15]. Sorafenib ameliorated bleomycin-induced pulmonary fibrosis through inhibiting EMT and fibroblast [16], and inhibited EMT in human lung epithelial cells [17]. Sorafenib was also used to suppress postsurgical recurrence and metastasis of HCC in an orthotopic mouse model, indicating that sorafenib had a potential application in early-stage HCC patients who have undergone hepatectomy with curative intention [18]. Apart from its successful application in patients with advanced, unresectable HCC, however, the use of sorafenib in patients with early-stage HCC is largely untested; this is especially true for patients who are considered as appropriate candidates for curative intervention. Whether sorafenib could be used to suppress the EMT of HCC after insufficient RFA and further prevent the progression of residual HCC remains poorly unknown.

In the present study, we established a simulated model to understand the recurrence of aggressive HCC after insufficient RFA. We investigated the effects of sorafenib on cell growth, migration and invasion of HCC cell lines (HepG2 and SMMC7721) after insufficient RFA *in vitro*. Furthermore, we analyzed the influences of sorafenib on changes of epithelial and mesenchymal markers, and Akt and ERK1/2 signaling pathways involved in the process in HCC cells after insufficient RFA. We also performed *in vivo* experiments to study the effect of sorafenib on the growth of HCC cells after insufficient RFA in a BALB/c nu/nu mice model.

## Methods

### Ethics statement

All animal experiments were approved by Animal Care Committee of Capital Medical University and performed in accordance with the institutional guideline. All sections of this report adhere to the ARRIVE Guidelines for reporting animal research [19]. A completed ARRIVE guidelines checklist is included in Additional file 1.

## Reagents

Sorafenib was kindly provided by Bayer Pharmaceuticals. 3-(4, 5-dimethylthiazol-2-yl)-2, 5-diphenyltetrazolium bromide (MTT) was obtained from Sigma (Shanghai, China). Phospho-anti-Akt and phospho-anti-ERK1/2 antibodies were purchased from Cell signaling (Beverly, CA, USA). Anti-E-cadherin, N-cadherin, vimentin, and snail were bought from Abcam (Cambridge, TX, USA). Anti-β-actin, PCNA, Ki67, MMP-2 and MMP-9 antibodies were obtained from Santa Cruz (Dallas, TX, USA). Enzyme-linked immunosorbent assay (ELISA) kits for E-cadherin, N-cadherin, MMP-2 and MMP-9 were purchased from RD (Minneapolis, MN, USA) and kits for vimentin and snail were from Bioss (Beijing, China).

### Cell culture

HCC cell lines, HepG2 and SMMC7721, were from the American Type Culture Collection (ATCC) (Manassas, VA, USA). HCC cells were cultured in high-glucose Dulbecco’s modified Eagle medium (DMEM) supplement with 10 % heat-inactivated fetal bovine serum (FBS), 100 U/ml penicillin and 100 μg/ml streptomycin (Life Technologies, Cergy Pontoise, France) at 37 °C in a humidified incubator with 5 % CO_2_.

### Heat treatment

Insufficient RFA was simulated *in vitro* as described before [11]. Briefly, HCC cells were seeded into the 6-well plates (5 × 10^4^ cells/well). After 24 h, the plates were sealed and submerged in a water bath set to 37 or 47 °C for 5 min. Thereafter, cells were allowed to recover, and when the surviving populations reached 80 % confluence, cells were propagated into the 6-well plates and exposed to above heat treatment for 10 min. Then the process was repeated and cells were sequentially exposed to above heat treatment for 15, 20 and 25 min. Cells surviving from the treatment of 47 °C for 25 min were designated as HepG2-H and SMMC7721-H cells.

### MTT assay

Cell viability was analyzed using the MTT assay. Briefly, HCC cells were cultured in 96-well plates at a concentration of 3 × 10^3^/well, incubated for 24 h, and treated with sorafenib. After 24, 48, or 72 h treatment, MTT solution was added to each well at a final concentration of 0.5 mg/ml and the cells were incubated for 4 h. At the end of incubation, formazan crystals resulting from MTT reduction were dissolved by addition of 150 μl dimethyl sulfoxide per well. The absorbance was measured at 570 nm using an automated ELISA plate reader.

### Colony formation assay

HCC cells (1 × 10^3^ /well) were seeded into 6-well dishes and allowed to grow for 24 h. The cells were then incubated in the presence or absence of sorafenib for 2 weeks in complete medium. The colonies obtained were washed with PBS and fixed in 4 % paraformaldehyde for 20 min at room temperature and then stained with crystal violet. The colonies were photographed under an inverted fluorescence microscope (Olympus IX51) equipped with an Olympus Qcolor 3 digital camera (Olympus). The colonies were counted from 6 random fields at 12.5 × magnification.

### Migration and invasion assays

Quantitative cell migration assays were performed using a modified Boyden chamber (Costar-Corning, New York, USA) with 8.0-μm pore polycarbonate filter inserts in 24-well plates as described previously. Briefly, the lower chamber was filled with DMEM with 10 % FBS, and HCC cells (5 × 10^4^ cells/well) in serum-free medium were added into the upper chamber. After 30 min, sorafenib was added to the upper chamber. The cells were allowed to migrate for 24 h at 37 °C. The non-migrated cells were removed from the upper surface of the membrane by scraping with a cotton swab, and the migrating cells were fixed with methanol, stained with crystal violet (Beyotime, Nantong, China) and photographed under an inverted fluorescence microscope (Olympus IX51) equipped with an Olympus Qcolor 3 digital camera (Olympus). Migration was assessed by counting the number of stained cells from 10 random fields at 200× magnification. Cell invasion assay was performed similarly, except that transwell inserts were matrigel-coated.

### Western blot analysis

HCC cells were lysed with lysis buffer [150 mM NaCl, 50 mM Tris-HCl (pH 8.0), 0.1 % SDS, 1 % Triton X-100] containing protease and phosphatase inhibitor. Cell lysate protein content was determined using a Bicinchoninic acid (BCA) protein assay kit. Equivalent amounts of whole cell extracts were subjected to SDS-PAGE gel and transferred to nitrocellulose membranes. The membranes were blocked with 5 % non-fat milk for 2 h and then incubated with respective primary antibody overnight at 4 °C followed by the incubation with the appropriate HRP-conjugated secondary antibody for 1.5 h at room temperature. Blots were visualized with an ECL detection kit (Pierce, USA) and analyzed using Quantity One 1-D Analysis Software (Bio-Rad, Hercules, USA).

### Enzyme-linked immunosorbent assay

Cytokines secreted into the conditioned medium were quantified using ELISA kits according to the manufacturer’s instructions. The concentration of cytokines was normalized to the total cellular protein using a BCA protein assay kit.

### Xenografts assay

BALB/C nude mice (male, 4–5 weeks old) were obtained from Vital River (Beijing, China). Mice were maintained under pathogen-free conditions and housed in barrier facilities on a 12-h light/dark cycle, with food and water ad libitum. HepG2 and HepG2-H cells (5 × 10^6^) were suspended in 200 μl serum-free DMEM and matrigel (1:1) and then injected subcutaneously into the upper right flank region of 20 nude mice. After 1 w, mice were treated with sorafenib by oral route (30 mg/kg/day), or polyoxyethylenated castor oil as control every day for up to the 22^th^ day (n = 5 each group). Tumor size was measured with a caliper rule every other day. The tumor volume was calculated as follows: TV (mm^3^) = (L × W^2^)/2, where L was the longest and W the shortest radius of the tumor in millimeters. At the end of the experiments, mice were euthanized by cervical dislocation, blood samples were collected via cardiac puncture after the mice were anesthetized i.p. with 400 mg/kg chloral hydrate (SCRC, Shanghai, China), and tumor tissues were removed for fixation in the 4 % paraformaldehyde for histologic examination and immunohistochemical staining.

### Immunohistochemical analysis

Tissues were fixed in 4 % paraformaldehyde and subsequently embedded in paraffin. Paraffin-embedded tissue sections were cut into standard 6 μm sections, deparaffinaged in xylene and rehydrated through graded alcohol solutions. Antigen retrieval was performed 10 min at 92 °C in EDTA (10 mM, pH 8.0) in a water bath. Endogenous peroxidases were inactivated by immersing the sections in 0.3 % hydrogen peroxide for 12 min. The sections were blocked with 5 % goat serum for 60 min at 37 °C. The slides were incubated with primary antibodies for overnight at 4 °C. Next, the slides were treated with appropriate HRP-conjugated secondary antibody for 40 min at 37 °C and then developed with 3,3’-diaminobenzidine. Finally, the slides were counterstained with hematoxylin and mounted. The slides were examined with Nikon Eclipse Ti microscope under a 200× objective.

### Statistical analysis

All values are expressed as the mean ± SEM. The data were analyzed using the ANOVA test. A P value of <0.05 was considered statistically significant. GraphPad Prism (GraphPad Software Inc., San Diego, California, USA) was used for these analyses.

## Results

### Sorafenib inhibited the enhanced viability of HCC cells after insufficient RFA

**Fig. 1:**
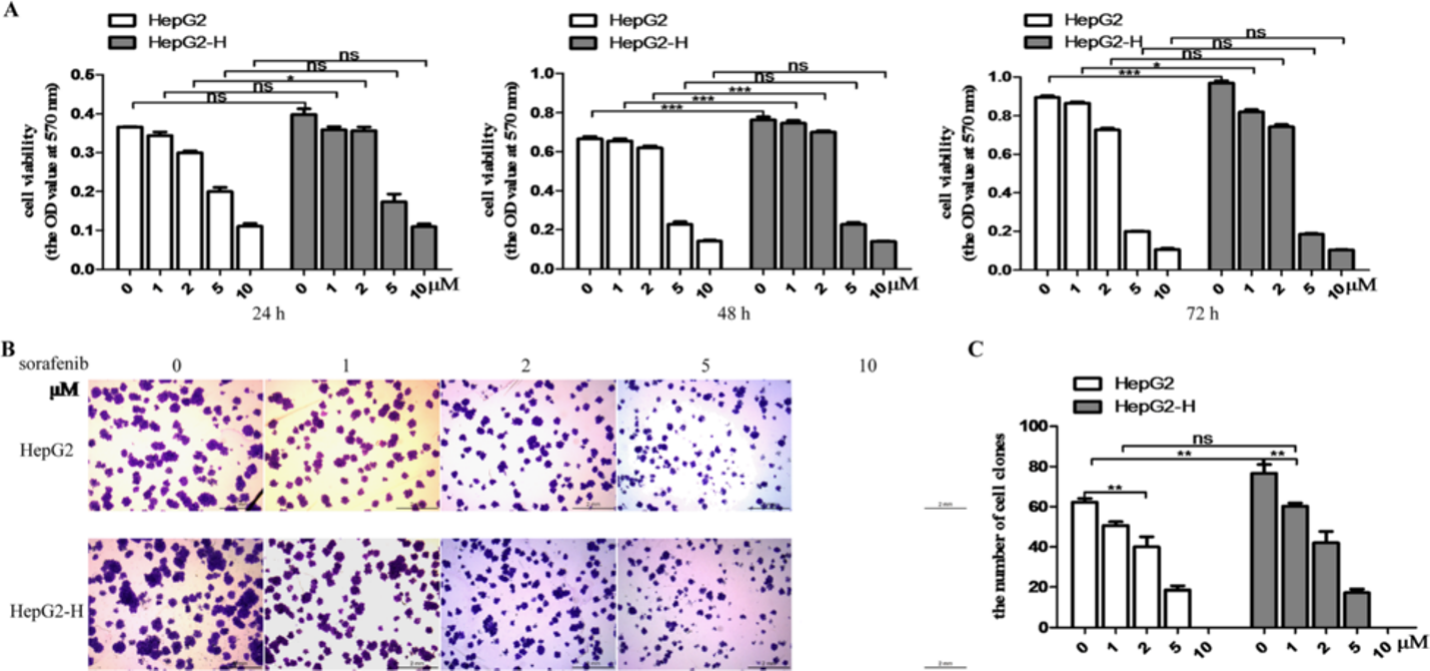
Sorafenib inhibited the enhanced viability of HCC cells after insufficient RFA. HepG2 cells were treated with insufficient RFA (47 °C 5 min, 10 min, 15 min, 20 min and 25 min) gradually. Residual HepG2 (named as HepG2-H) cells surviving from the treatment of 47 °C for 25 min were collected and used for the next experiments. (**a**) The effect of sorafenib on viability rate of HepG2 and HepG2-H cells was evaluated by MTT assay. Error bars represent the SEM of data obtained in five independent experiments. (**b** and **c**) Colony formation abilities of HepG2 and HepG2-H cells after the treatment of sorafenib were assessed. Representive images of the colonies were shown (12.5×) Error bars represent the SEM of data obtained in three independent experiments. *P* value <0.05 was considered statistically significant; **p < 0.01, ***p < 0.001

First of all, HepG2 or SMMC7721 cells were treated with heat treatment for 5, 10, 15, 20 and 25 min gradually as described above. Cells surviving from the treatment of 47 °C for 25 min were designated as HepG2-H and SMMC7721-H cells when we could see the morphological changes. To evaluate the effects of sorafenib on HCC cells, all cells were treated with sorafenib for 24, 48 or 72 h at different concentrations. HepG2-H cells exhibited higher viability rate compared with HepG2 cells at 48 and 72 h (Fig. 1a). Sorafenib inhibited the viability rate of HepG2 and HepG2-H cells in a time- and dose- dependent manner (Fig. 1a). After the treatment of sorafenib (5 and 10 μM) for 24 and 48 h and sorafenib (2, 5 and 10 μM) for 72 h, the distinction of viability rate between HepG2 and HepG2-H cells faded (Fig. 1a). To determine the long term growth ability, sorafenib was allowed to treated HCC cells for 2 weeks. HepG2-H cells had a higher number of colonies in comparison with HepG2 cells (Fig. 1b and c). Sorafenib also suppressed the colony formation of HepG2 and HepG2-H cells in a dose-dependent manner. Similar results were also found in SMMC7721 and SMMC7721-H cells (Additional file 2: Figure S1).

### Sorafenib inhibited the enhanced migration and invasion abilities of HCC cells after insufficient RFA

**Fig. 2:**
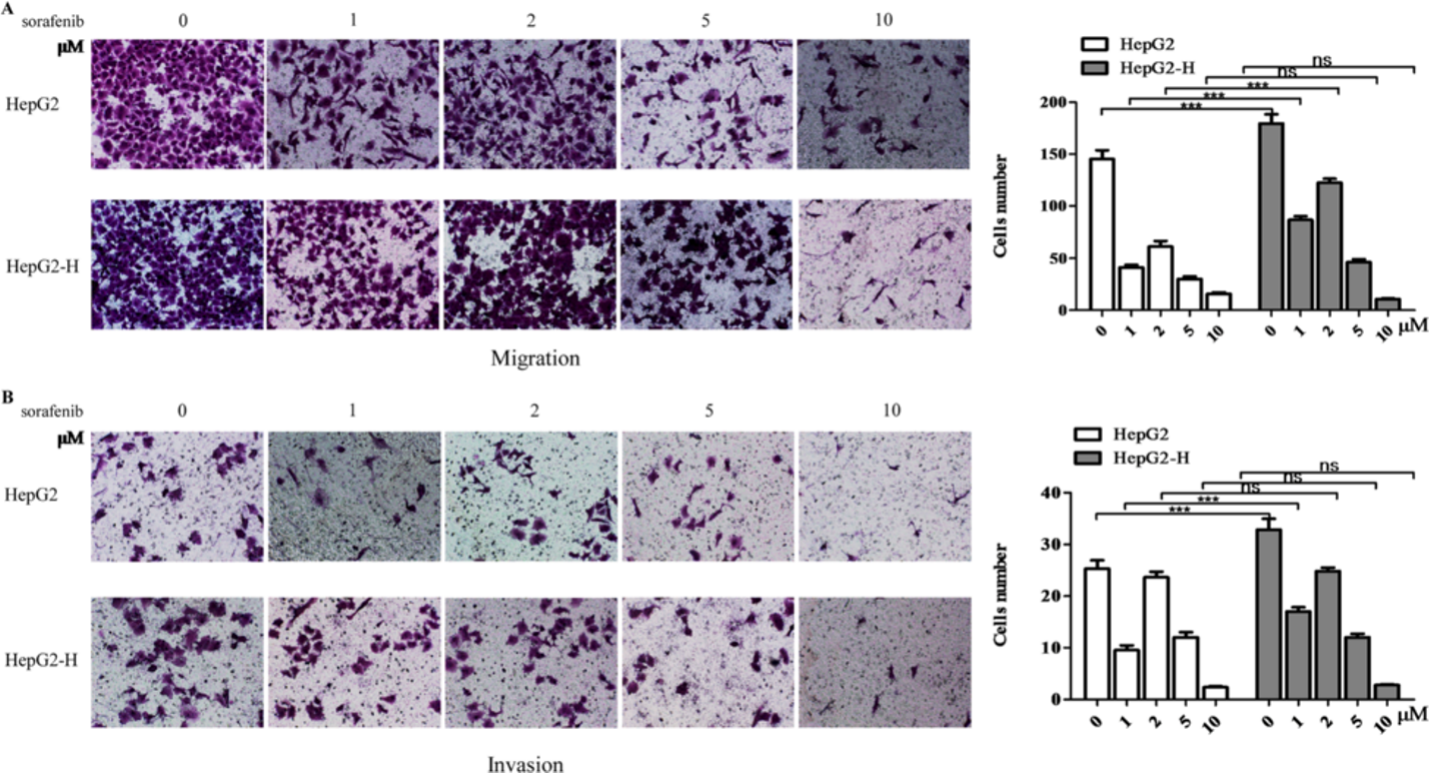
Sorafenib inhibited the enhanced migration and invasion abilities of HCC cells after insufficient RFA. The effects of sorafenib on migration (**a**) and invasion (**b**) of HepG2 and HepG2-H cells were shown. Error bars represent the SEM of data obtained in three independent experiments. *P* value <0.05 was considered statistically significant; **p < 0.01, ***p < 0.001

HepG2-H cells displayed enhanced migration and invasion abilities compared with HepG2 cells (Fig. 2). Sorafenib inhibited the migration and invasion abilities of HepG2 and HepG2-H cells (Fig. 2). Sorafenib (5 and 10 μM) eliminated the difference of migration ability between HepG2 and HepG2-H cells, and sorafenib (2, 5 and 10 μM) eliminated the difference of invasion ability between HepG2 and HepG2-H cells (Fig. 2). Similar results were also in SMMC7721 and SMMC7721-H cells (Additional file 2: Figure S2).

### Sorafenib suppressed the EMT of HCC cells after insufficient RFA

**Fig. 3:**
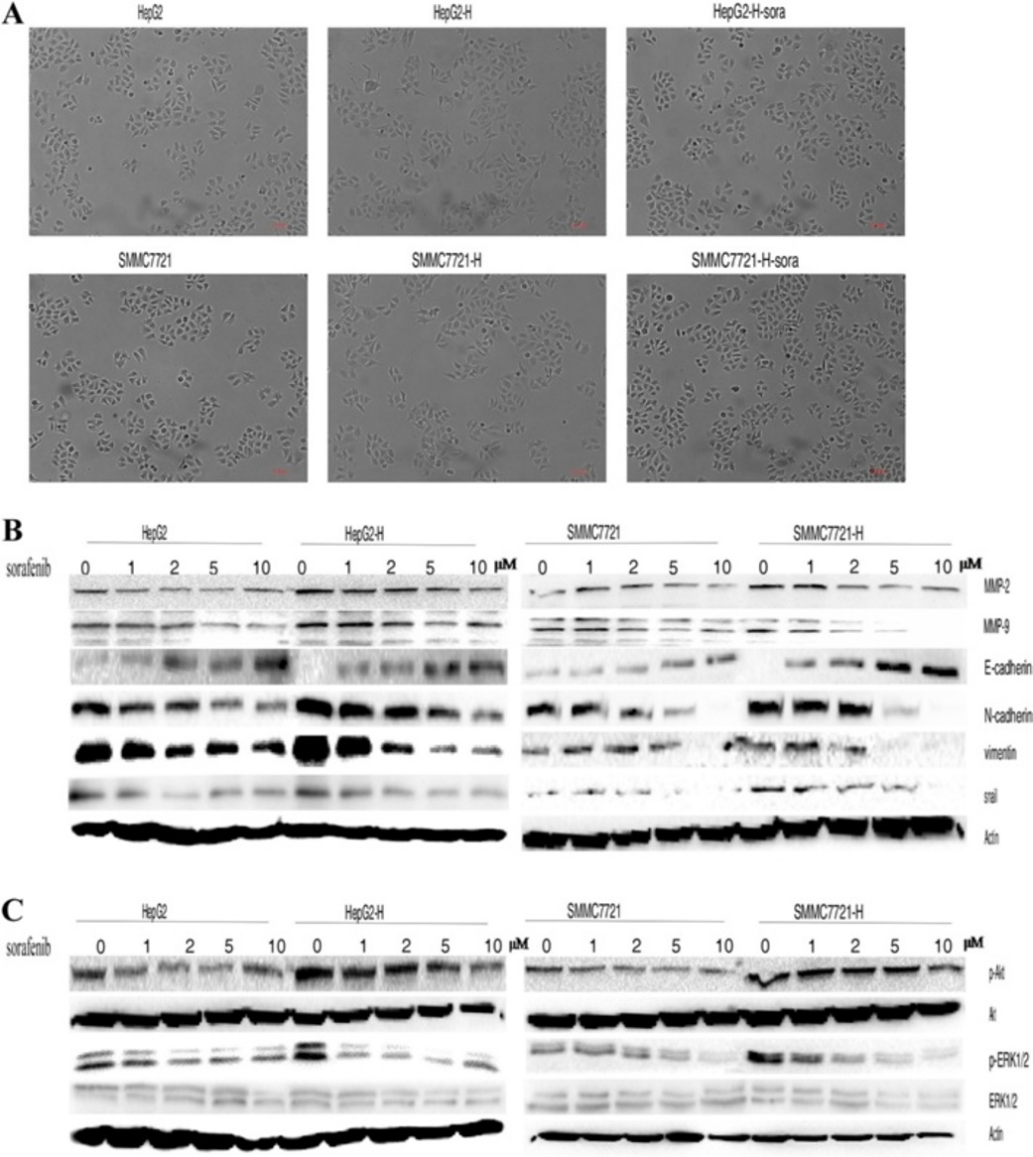
Sorafenib suppressed the EMT of HCC cells after insufficient RFA. Sorafenib was used to treat HCC cells, the morphological changes of HepG2 and SMMC7721 after insufficient RFA were displayed (**a**) and western blot was used to determine the expression of MMP-2, MMP-9, E-cadherin, N-cadherin, vimentin, snail (**b**), p-Akt, Akt, p-ERK1/2 and ERK1/2 (**c**)

**Fig. 4:**
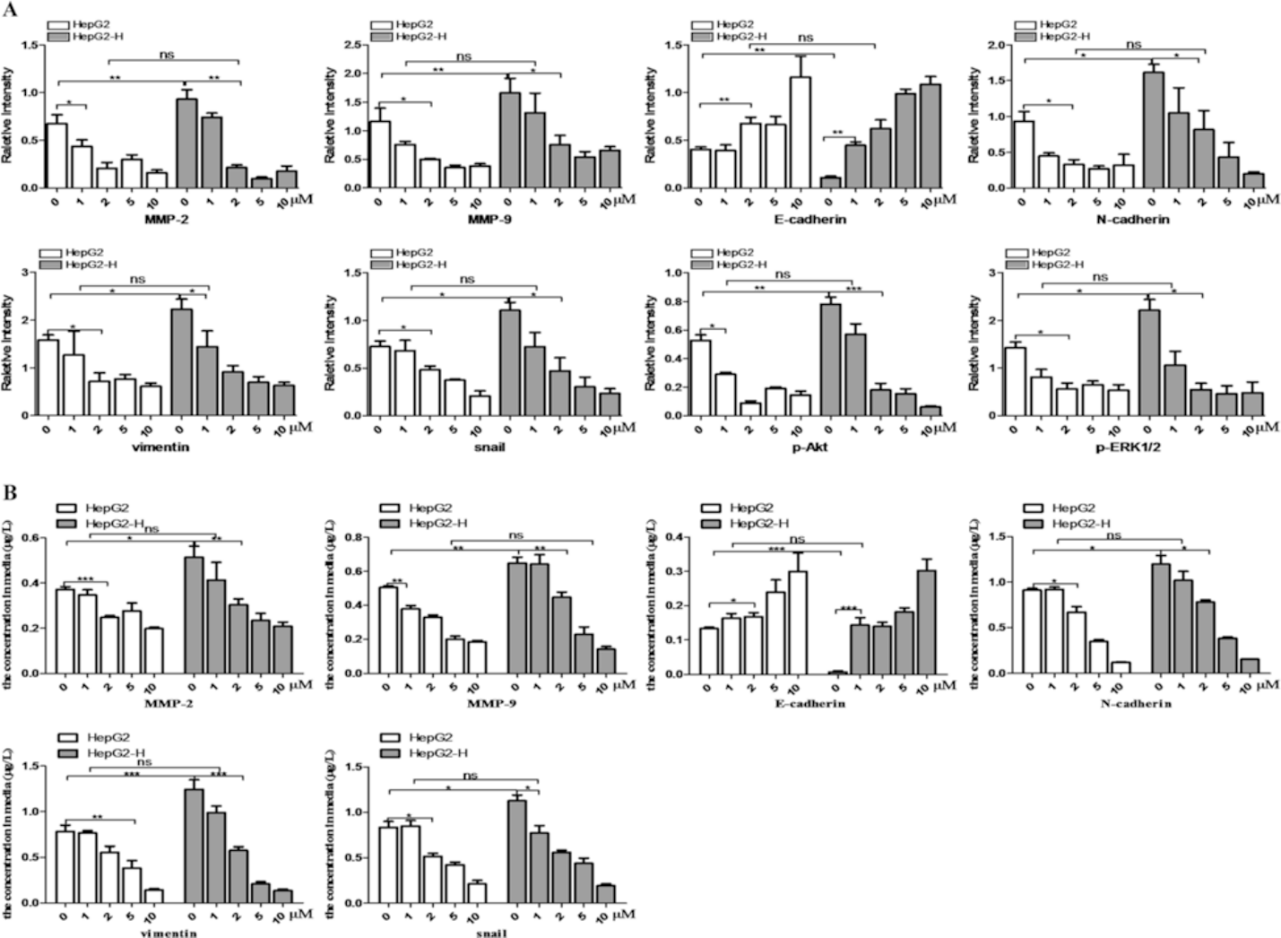
Sorafenib suppressed the EMT of HCC cells after insufficient RFA. Gray analysis of all bands was used to quantify expression levels of MMP-2, MMP-9, E-cadherin, N-cadherin, vimentin, snail, p-Akt, Akt, p-ERK1/2 and ERK1/2 in HepG2 and HepG2-H cells (**a**) The concentration of cytokines secreted into the conditioned medium of HepG2 and HepG2-H cells was detected by ELISA analysis (**b**). Error bars represent the SEM of data obtained in three independent experiments. *P* value <0.05 was considered statistically significant; **p < 0.01, ***p < 0.001

HepG2-H and SMMC7721-H displayed a spindle shape with less cell-cell adhesion and increased formation of pseudopodia, and sorafenib inhibited the morphological changes above (Fig. 3a). To evaluate whether EMT had occurred in HepG2-H and SMMC7721-H cells, EMT markers were examined. Western blot and ELISA assay showed that significant reduction in E-cadherin expression and up-regulation of N-cadherin, vimentin, snail, MMP-2 and MMP-9 were found in HepG2-H (Figs. 3b and 4) and SMMC7721-H cells (Fig. 3b and Additional file 2: Figure S3). Sorafenib increased the expression of E-cadherin, and decreased the expression of N-cadherin, vimentin, snail, MMP-2 and MMP-9 in HCC cells in a dose-dependent manner (Figs. 3b, 4 and Additional file 2: Figure S3). To explore the signaling mechanisms involved in the sorafenib on EMT of HCC cells after insufficient RFA, we tested Akt and ERK1/2 signaling pathways. HepG2-H and SMMC7721-H cells showed significantly increased expression of p-Akt and p-ERK1/2 compared with HepG2 and SMMC7721 cells respectively (Figs. 3c, 4a and Additional file 2: Figure S3A). Furthermore, sorafenib inhibited the expression of p-Akt and p-ERK1/2 in HCC cells in a dose-dependent manner (Figs. 3c, 4a and Additional file 2: Figure S3A).

### Insufficient RFA enhanced the growth of HCC cells *in vivo*, and sorafenib blocked the process

**Fig. 5:**
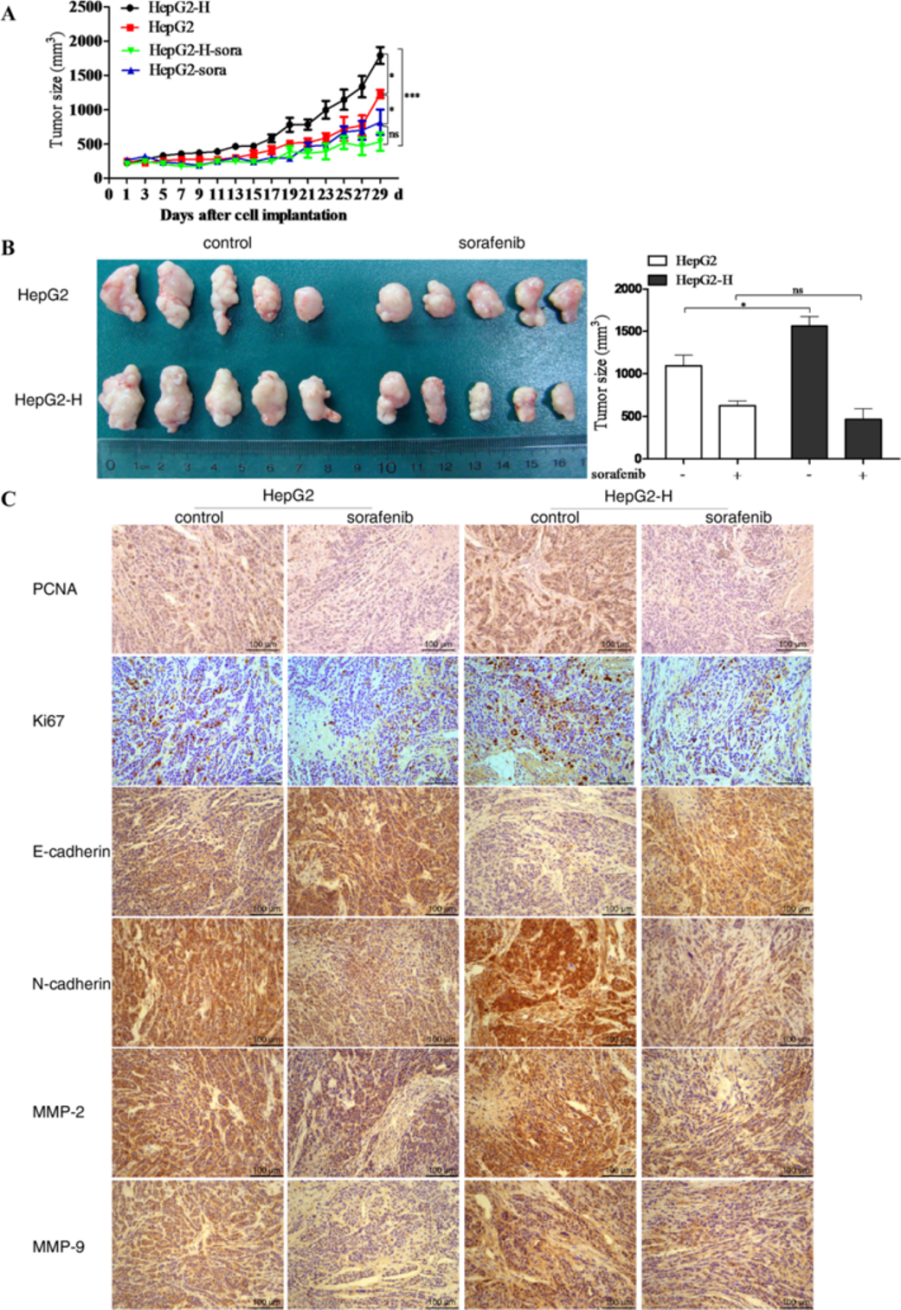
Insufficient RFA enhanced the growth of HCC cells *in vivo*, and sorafenib blocked the process. HepG2 and HepG2-H cells were injected subcutaneously into the upper right flank region of nude mice, treated with or without sorafenib, and tumor volume was measured. (**a**) Tumor volume was measured with a caliper rule every other day. Data were presented as the mean tumor volumes of mice. (**b**) Tumor size of the 29^th^ day was displayed. (**c**) Tumor sections were stained for PCNA, Ki67, MMP-2, MMP-9, E-cadherin and N-cadherin. Representive images of the immunohistochemistry assay were shown (200×)

HepG2-H cells showed increased tumor volume compared with HepG2 cells (Fig. 5a and b). Sorafenib suppressed the growth of HepG2 and HepG2-H cells (Fig. 5a and b). Sorafenib diminished the difference of tumor growth between HepG2 and HepG2-H cells (Fig. 5a and b). Significant increases of cell proliferation were observed in HepG2-H cells, and sorafenib inhibited the process (Fig. 5c). In addition, HepG2-H tumors showed decreased expression of E-cadherin and increased expression of N-cadherin, MMP-2 and MMP-9 compared with HepG2 tumors (Fig. 5c). And sorafenib increased the expression of E-cadherin, and decreased the expression of N-cadherin, MMP-2 and MMP-9 in HepG2 and HepG2-H tumors (Fig. 5c). However, there were no apparent changes in liver, heart, kidney, lung and body weight in the mice (Additional file 2: Figure S4).

## Discussion

Local recurrences of HCC can progress rapidly after RFA, and EMT may involve in the progress. In the present study, we established a model simulating the HCC cells after insufficient RFA. And, we showed that sorafenib significantly suppressed the EMT of HCC after insufficient RFA *in vitro* and *vivo*. The finding may have a beneficial impact on the clinical practice of HCC therapy and especially on the radical intervention used for early-stage patients. Sorafenib may be applied to patients who have undergone RFA, and used to improve the prognosis via the effective inhibition of EMT.

Our previous study demonstrated that EMT occurred in SMMC7721 and Huh7 cells after insufficient RFA [11]. In this study, HepG2 cells were treated with insufficient RFA gradually as previous, and EMT also occurred. In another research, HepG2, Huh7 and HepG3B cells were exposed to 50 °C for 10 min, and EMT occurred on day 5 after treatment, which was partly consistent with our results [12]. Our results further confirmed that insufficient RFA promoted the EMT of HCC cells. Furthermore, in our results, sorafenib suppressed the EMT of HCC cells after insufficient RFA, which was not ever reported before.

Recently, Akt and ERK signaling pathways have been reported to play an important role in EMT in HCC. MicroRNA-331-3p promoted proliferation and EMT-mediated metastasis of HCC via the suppression of PHLPP-mediated de-phosphorylation of Akt [20]. Maelstrom promoted HCC metastasis by inducing EMT by way of Akt/GSK-3β pathways [21]. MicroRNA-21 suppressed PTEN and hSulf-1 expression and promoted HCC progression through Akt/ERK pathways [22]. Akt and ERK pathways were also mediated the EMT of HCC cells after insufficient RFA [11], and sublethal heat treatment promoted EMT of HCC via ERK pathways [12]. Therefore, the inhibitors of Akt and ERK may be applied to suppress the EMT of HCC cells after insufficient RFA. However, all specific inhibitors of Akt are in preclinical trials [23, 24]. Sorafenib was originally developed based on its inhibitory effect of Raf and receptor tyrosine kinase signaling, which further inhibited the Raf-MEK-ERK signaling pathway [25]. Sorafenib has been reported to suppress the growth and metastasis of HCC via Akt and ERK pathways [26–28], and inhibit EMT of HCC induced by growth factors [14, 15]. In the present study, sorafenib inhibited the up-regulation of p-Akt and p-ERK1/2 in HCC cells after insufficient RFA, and further down-regulated the increased expression of N-cadherin, vimentin and snail, which resulted in enhanced abilities of migration and invasion in HCC cells after insufficient RFA.

Recently, sorafenib has been used to strengthen the curative effect of RFA in HCC. Fukuda H et al. reported that combination of sorafenib and RFA may be superior to standard RFA alone in the treatment of HCC tumors smaller than 3 cm in diameter [29]. Li Y et al. reported that the combination of sorafenib, transarterial chemoemboliztion and RFA proved both safe and effective in the treatment for unresectable HCC patients [30]. Another research demonstrated that sorafenib suppressed the rapid progress of HCC after insufficient ablation therapy in an experiment *in vivo*, which seemed like our research [31]. In their study, sorafenib was able to inhibit the up-regulated expression of HIF-1α and VEGFA in a xenograft model after partial RFA. However, rapid progress of HCC after insufficient RFA was not observed, and further mechanisms involved in the progress were not investigated. According to the guidelines of the National Centre for the Replacement, Refinement and Reduction of Animals in Research (London, UK), in the animal experiment, we produced reasonable evidence to support our findings *in vitro* by stopping large studies which would result in a large augment in animal use. In our study, sorafenib inhibited the migration and invasion of HCC cells through the suppression of EMT after insufficient RFA in the simulated model. Furthermore, in the mice model, HCC cells after insufficient RFA exhibited larger tumor volume compared with HCC cells after control treatment, and sorafenib inhibited the growth of HCC cells and eliminated the difference of growth in HepG2-H and HepG2 cells. So sorafenib may be used to prevent the progression of HCC cells after insufficient RFA.

Sorafenib causes multiple human toxicities, including use-limiting anorexia, GI bleeds and hand-foot syndrome, which may lead to drug discontinuation or dose reduction that can decrease the potential life-prolonging benefits of sorafenib [32, 33]. In our research, sorafenib (5 μM) significantly suppressed the enhanced abilities of migration and invasion of HCC cells associated with EMT after insufficient RFA *in vitro*, and sorafenib (30 mg/kg/day) inhibited the growth of HCC cells after insufficient RFA *in vivo* without more toxicity for mice. However, in the present study, we just used the simulated model which may not be representative of the progression of residual HCC tumor after insufficient RFA effectively and only provided the preliminary basis for the application of sorafenib to prevent the progression of HCC cells after RFA, and more mechanisms involved in the progression and clinical trials should be investigated in the future.

## Conclusions

All in all, the study focused on a simulated model to understand the recurrence of aggressive HCC after insufficient RFA. This study demonstrated that insufficient RFA promoted the EMT of HCC cells. Sorafenib inhibited the EMT of HCC cells after insufficient RFA, and may be used to prevent the progression of HCC after RFA.

## Additional files

Additional file 1: **NC3Rs ARRIVE Guidelines Checklist.** (DOCX 664 kb)
Additional file 2: **The supplementary data.** (DOCX 4641 kb)

## Abbreviations

BCA: Bicinchoninic acid
DMEM: Dulbecco’s modified Eagle medium
ELISA: Enzyme-linked immunosorbent assay
EMT: Epithelial-mesenchymal transition
FBS: fetal bovine serum
HCC: hepatocellular carcinoma
MTT: 3-(4, 5-dimethylthiazol-2-yl)-2, 5-diphenyltetrazolium bromide
RFA: insufficient radiofrequency ablation
