# Supplementary material for: Sorafenib suppresses the epithelial-mesenchymal transition of hepatocellular carcinoma cells after insufficient radiofrequency ablation
Source: BMC Cancer. 2015 Nov 30;15:939. doi: 10.1186/s12885-015-1949-7 (PMC4663721; doi:10.1186/s12885-015-1949-7)

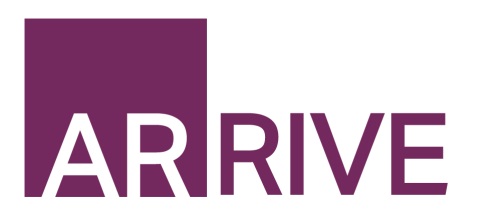


The ARRIVE Guidelines Checklist

Animal Research: Reporting In Vivo Experiments

Carol Kilkenny^1^, William J Browne^2^, Innes C Cuthill^3^, Michael Emerson^4^ and Douglas G Altman^5^

*^1^The National Centre for the Replacement, Refinement and Reduction of Animals in Research, London, UK, ^2^School of Veterinary Science, University of Bristol, Bristol, UK, ^3^School of Biological Sciences, University of Bristol, Bristol, UK, ^4^National Heart and Lung Institute, Imperial College London, UK, ^5^Centre for Statistics in Medicine, University of Oxford, Oxford, UK.*

|  | | ITEM | RECOMMENDATION | Section/ Paragraph |
| --- | --- | --- | --- | --- |
| 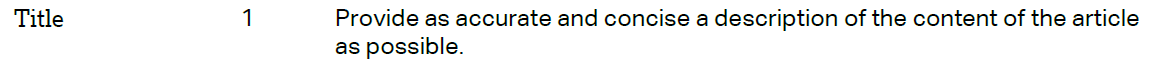 | | | Title |  |
| 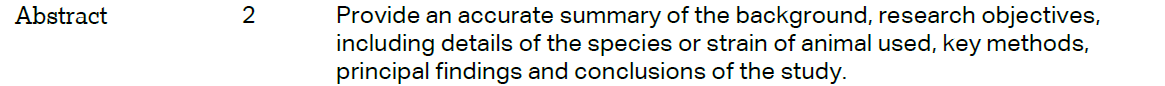 | | | Abstract |  |
| INTRODUCTION | | |  |  |
| 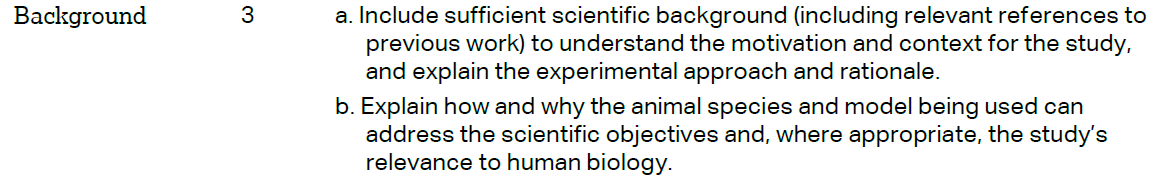 | | | Paragraphs 1-4  Paragraphs 2-4 |  |
| 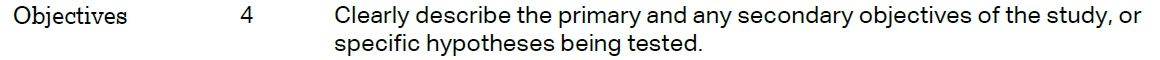 | | | Paragraph 4 |  |
| METHODS | | |  |  |
| 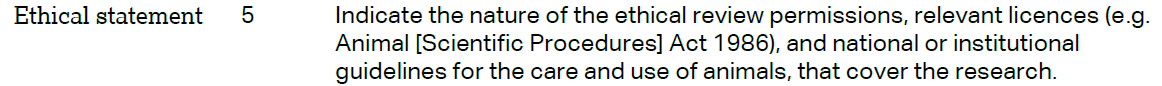 | | | Paragraph 1 |  |
| 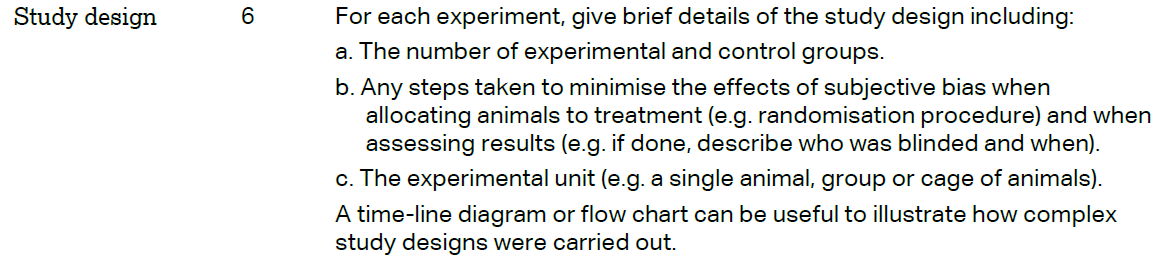 | | | Paragraph 10 |  |
| 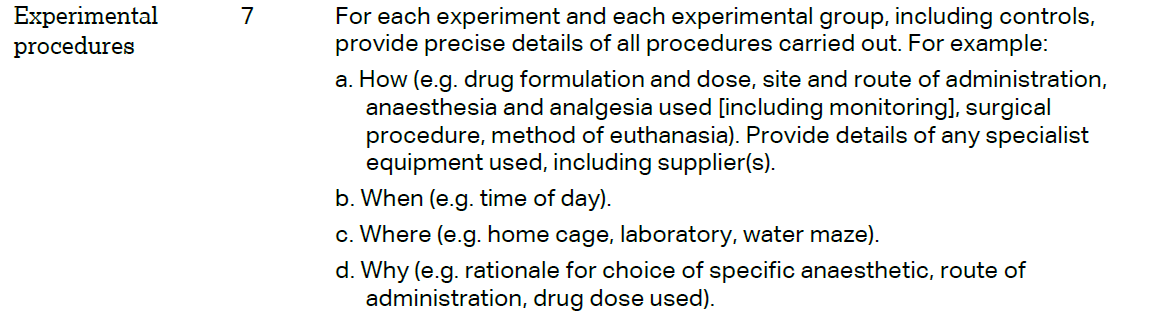 | | | a. b. d.  Paragraph 10  c. laboratory |  |
| 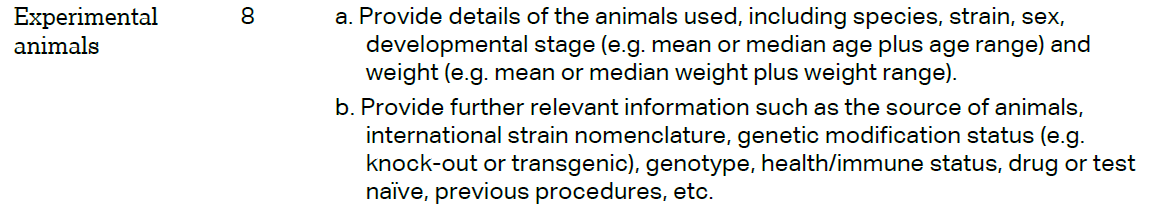 | | | Paragraph 10 |  |

The ARRIVE guidelines. Originally published in *PLoS Biology*, June 2010^1^

| 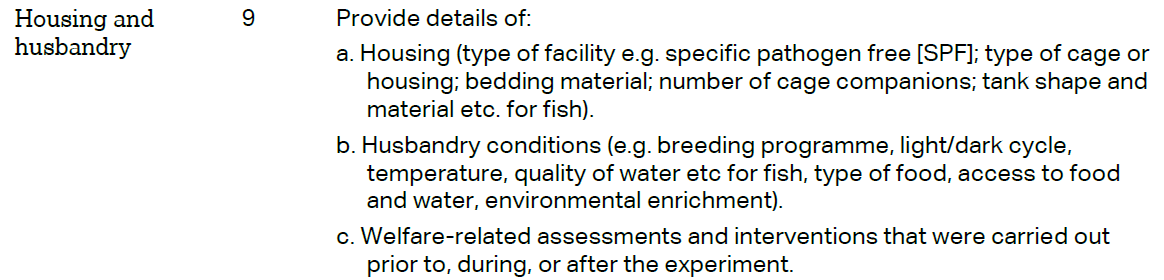 | Paragraph 10 | |
| --- | --- | --- |
| 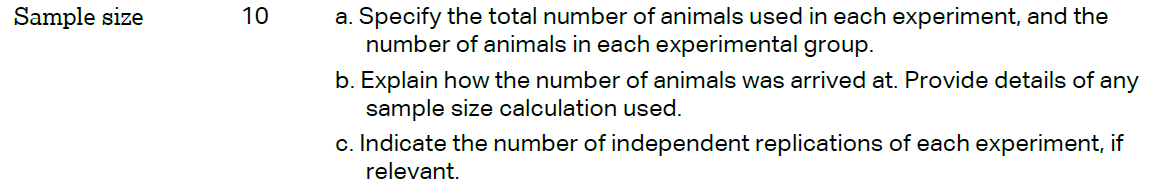 | a.  Paragraph 10  b. c.  Based on the minimal representative number and the 3Rs | |
| 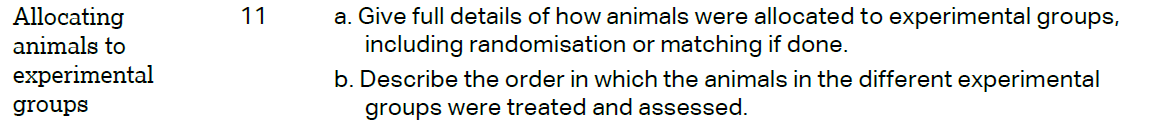 | Paragraph 10 | |
| 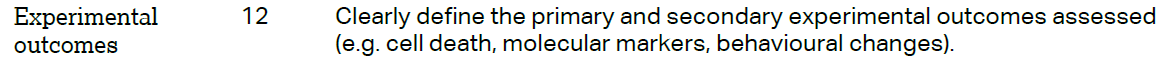 | Paragraph 10 | |
| 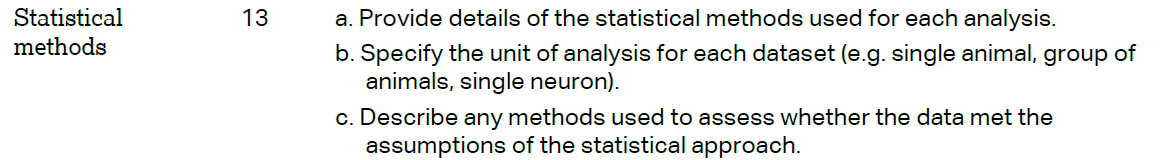 | Paragraph 10  and Figure 5 | |
| RESULTS |  | |
| 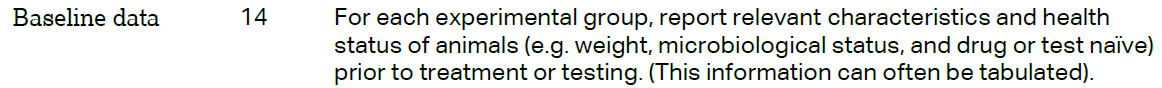 | Methods  Paragraph 4 | |
| 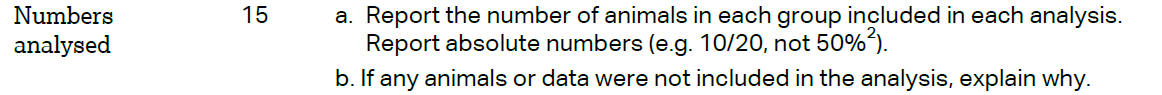 | Paragraph 4  and Figure 5 & Figure S4 | |
| 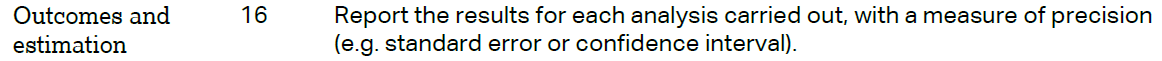 | Paragraph 4  and Figure 5 & Figure S4 | |
| 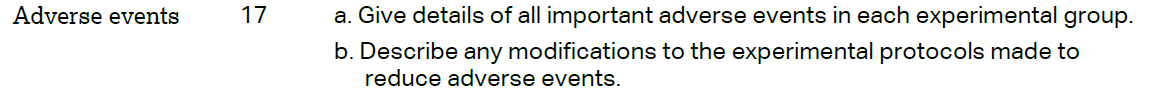 | Paragraph 4  and Figure S4 | |
| DISCUSSION |  | |
| 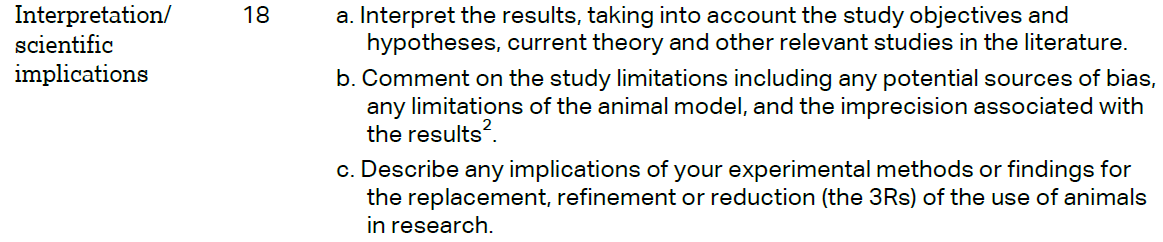 | Paragraph 4 & 5  Paragraph 5  Paragraph 4 | |
| 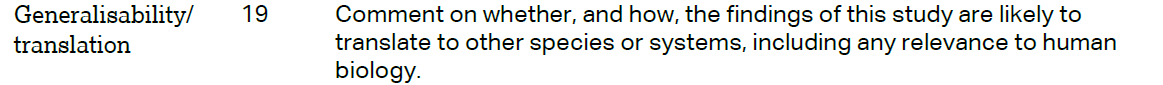 | Paragraph 1 4 5 | |
| 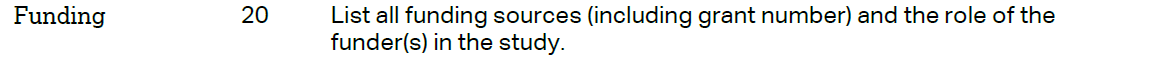 | | Paragraph 9 |


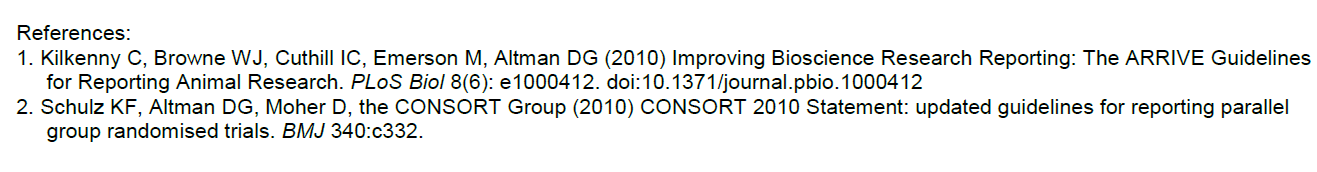

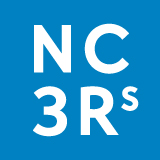

Supplement: Additional file 1: — NC3Rs ARRIVE Guidelines Checklist. (DOCX 664 kb) [file 12885_2015_1949_MOESM1_ESM.docx]
