# Supplementary material for: Sorafenib suppresses the epithelial-mesenchymal transition of hepatocellular carcinoma cells after insufficient radiofrequency ablation
Source: BMC Cancer. 2015 Nov 30;15:939. doi: 10.1186/s12885-015-1949-7 (PMC4663721; doi:10.1186/s12885-015-1949-7)

**NC3Rs ARRIVE Guidelines Checklist: (The checklist could be seen in Additional file 1).** (DOC 664 KB)

Format: DOC Size: 664KB

**Additional file 2**

**Figure S1.:** Sorafenib inhibited the enhanced viability of HCC cells after insufficient RFA. SMMC7721 cells were treated with insufficient RFA (47℃ 5 min, 10 min, 15 min, 20 min and 25 min) gradually. Residual SMMC7721 (named as SMMC7721-H) cells surviving from the treatment of 47℃for 25 min were collected and used for the next experiments. (A) The effect of sorafenib on viability rate of SMMC7721 and SMMC7721-H cells was evaluated by MTT assay. Error bars represent the SEM of data obtained in five independent experiments. (B) Colony formation abilities of SMMC7721 and SMMC7721-H cells after the treatment of sorafenib were assessed. Representive images of the colonies were shown (12.5×). Error bars represent the SEM of data obtained in three independent experiments. P value <0.05 was considered statistically significant; *p<0.05, **p<0.01, ***p<0.001. (TIFF 2.61 MB)

Format: TIFF Size: 2.61 MB

**Figure S2.:** Sorafenib inhibited the enhanced migration and invasion abilities of HCC cells after insufficient RFA. The effect of sorafenib on migration (A and C) and invasion (B and D) of SMMC7721 and SMMC7721-H cells were shown. Error bars represent the SEM of data obtained in three independent experiments. P value <0.05 was considered statistically significant; *p<0.05, **p<0.01. (TIFF 6.57 MB)

Format: TIFF Size: 6.57 MB

**Figure S3.:** Sorafenib suppressed the EMT of HCC cells after insufficient RFA. Gray analysis of all bands was used to quantify expression levels of MMP-2, MMP-9, E-cadherin, N-cadherin, vimentin, snail, p-Akt, Akt, p-ERK1/2 and ERK1/2 in SMMC7721 and SMMC7721-H cells (A).The concentration of cytokines secreted into the conditioned medium of SMMC7721 and SMMC7721-H cells was detected by ELISA analysis (B). Error bars represent the SEM of data obtained in three independent experiments. P value <0.05 was considered statistically significant; **p<0.01, ***p<0.001. (TIFF 221 KB)

Format: TIFF Size: 221 KB

**Figure S4.:** No apparent changes in liver, heart, kidney, lung and body weight in nude mice bearing with tumor. HepG2 and HepG2-H cells were injected subcutaneously into the upper right flank region of nude mice, and treated with or without sorafenib. (A) Mice weight was measured with a scale every other day. (B) Heart, lung, liver and kidney sections were stained with haematoxylin and eosin (HE). Representive images were shown (200×). (TIFF 7.79 MB)

Format: TIFF Size: 7.79 MB


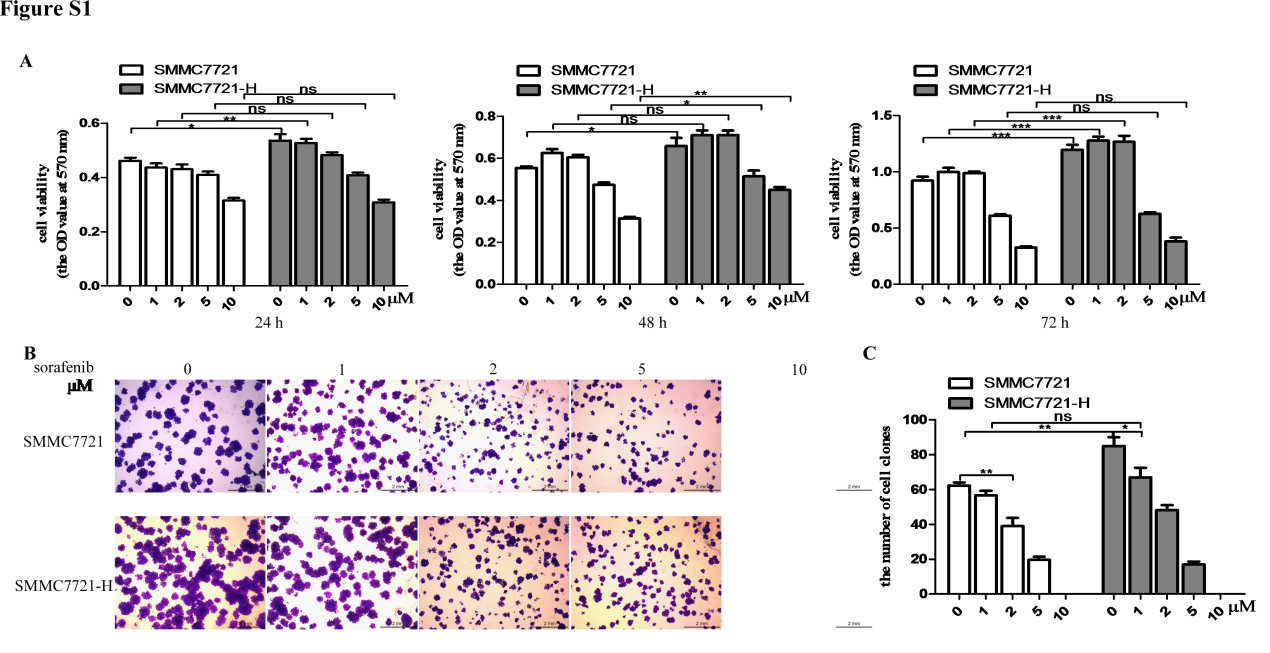


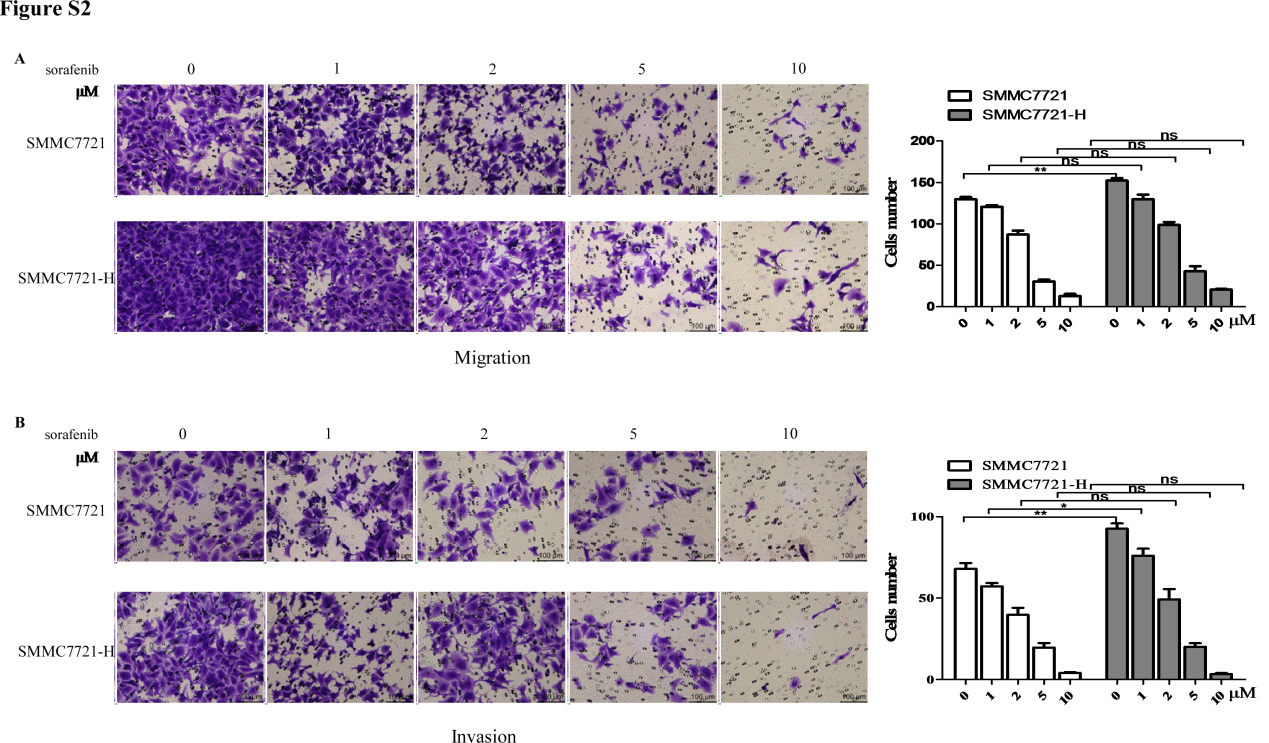


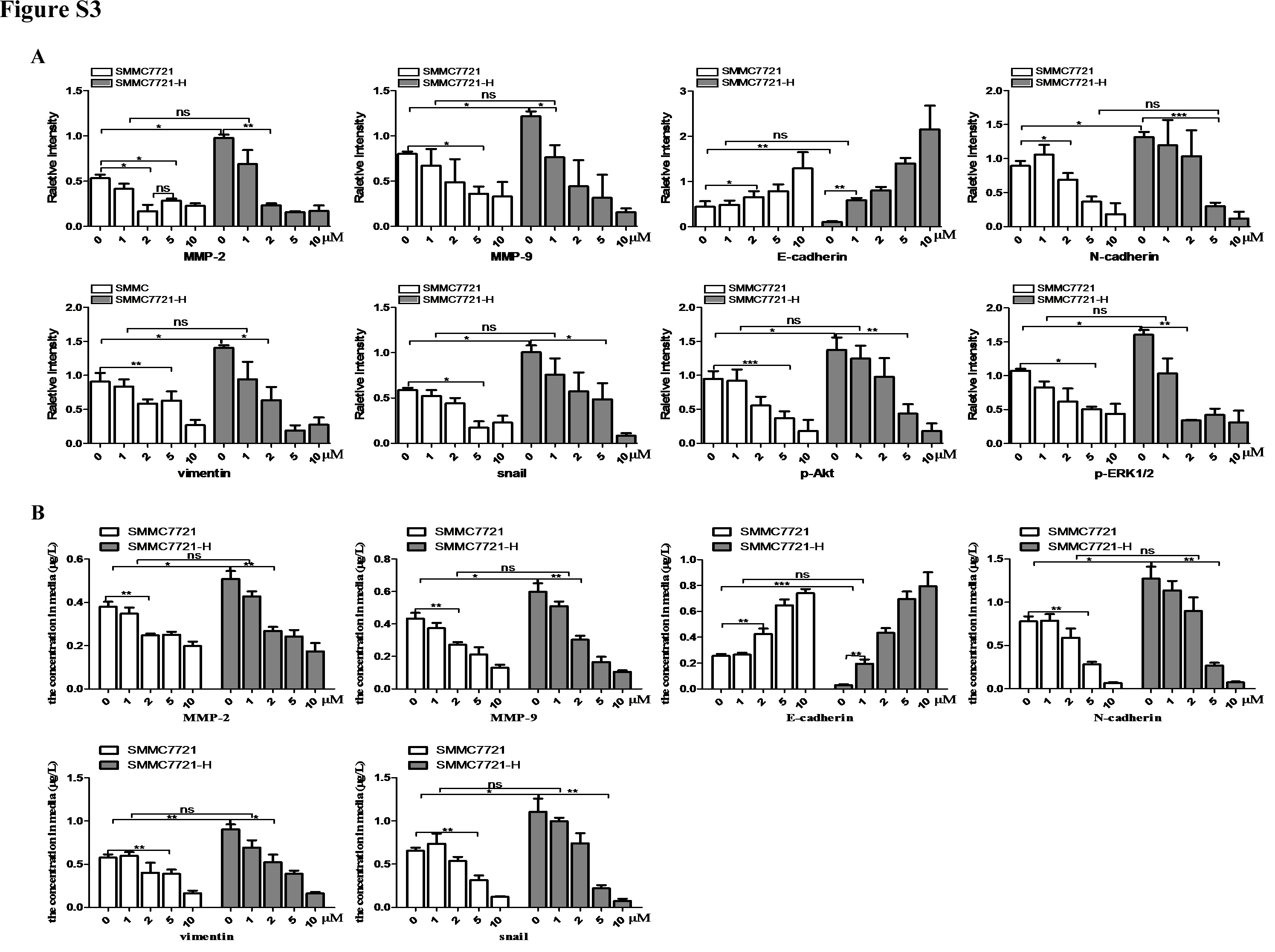


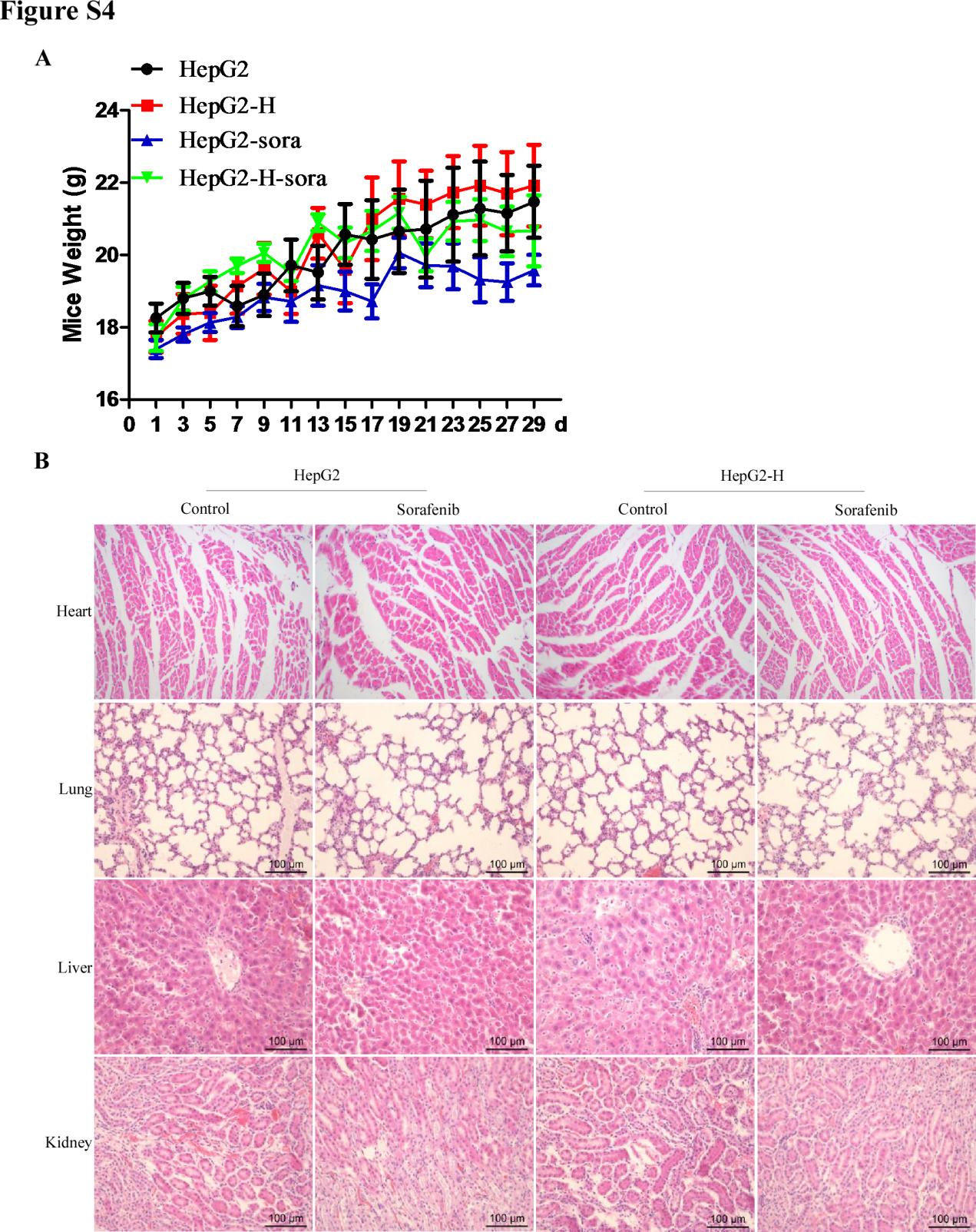

Supplement: Additional file 2: — The supplementary data. (DOCX 4641 kb) [file 12885_2015_1949_MOESM2_ESM.docx]
